# Bayesian inference of polymerase dynamics over the exclusion process

**DOI:** 10.1098/rsos.221469

**Published:** 2023-08-02

**Authors:** Massimo Cavallaro, Yuexuan Wang, Daniel Hebenstreit, Ritabrata Dutta

**Affiliations:** ^1^ Mathematics Institute, University of Warwick, Coventry, UK; ^2^ School of Life Sciences, University of Warwick, Coventry, UK; ^3^ Zeeman Institute for Systems Biology and Infectious Disease Epidemiology Research, University of Warwick, Coventry, UK; ^4^ Department of Statistics, University of Warwick, Coventry, UK; ^5^ Institute of Applied Statistics, Johannes Kepler Universität, Linz, Austria

**Keywords:** gene expression, non-equilbrium physics, Bayesian statistics, particle transport

## Abstract

Transcription is a complex phenomenon that permits the conversion of genetic information into phenotype by means of an enzyme called RNA polymerase, which erratically moves along and scans the DNA template. We perform Bayesian inference over a paradigmatic mechanistic model of non-equilibrium statistical physics, i.e. the asymmetric exclusion processes in the hydrodynamic limit, assuming a Gaussian process prior for the polymerase progression rate as a latent variable. Our framework allows us to infer the speed of polymerases during transcription given their spatial distribution, while avoiding the explicit inversion of the system’s dynamics. The results, which show processing rates strongly varying with genomic position and minor role of traffic-like congestion, may have strong implications for the understanding of gene expression.

## 1. Introduction

DNA is a long polymeric molecule that encodes information as a sequence of nucleotides (Nts). Turning this information into a phenotype is a complex phenomenon hinged upon transcription, the molecular process in which particular segments of DNA (i.e. the genes) are scanned and their information is copied into mRNA by the enzyme RNA polymerase II (PolII). The transcription itself consists of several steps which can be differentially regulated to alter the timing and the output of the mRNA production [1,2].

**Figure 1.**
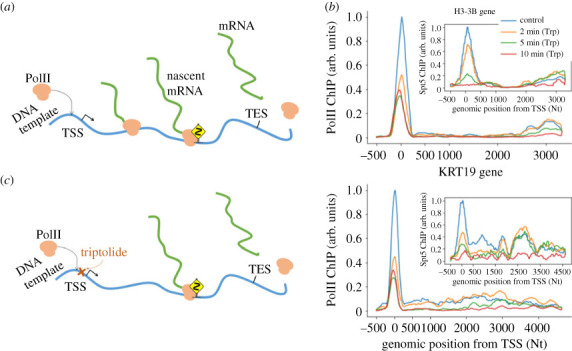
Biological processes and data. (*a*) Simplified diagram of mRNA synthesis. PolII molecules bind to the DNA upstream of TSS and moves downstream towards the TES, where it is released along with the synthesized mRNA. In certain genomic regions (indicated by a dangerous bend sign), PolIIs slow down. (*b*) ChIP-seq experiments yield the relative abundance of PolII at each genomic position, here illustrated for *H3-3B* (top) and *KR19* (bottom) genes; insets show the Spt5-bound PolII abundances for the same genes. (*c*) In the presence of triptolide (Trp), transport is blocked upstream of TSS, while transcriptional engaged PolII are allowed to complete elongation; this is reflected in the ChIP-seq profiles obtained 2, 5 and 10 min after treatment (also in (*b*)).

The transcription can also be seen as a non-equilibrium process, where the PolIIs are being transported as particles on a one-dimensional lattice, the lattice being the DNA template which the PolIIs bind to. We can further consider this process having left and right boundaries, representing the transcription start site (TSS) and the transcription end site (TES), respectively (figure 1*a*). Within the gene body, the PolIIs erratically travel along the template and their abrupt slowing down in certain genomic regions is known as *pausing* dynamics [3,4]. While the pausing is an essential part of the transcriptional machinery and contributes to the regulation of genes’ expression levels, a comprehensive quantitative understanding of its dynamics is still missing [5,6].

We present a modelling framework to help understand gene regulation and quantitatively study the pausing dynamics given real-world data. In the literature, a number of different mechanistic models have been introduced to elucidate transcription, starting from the simple telegraph model [7] to more complicated multi-state models that account for many interactions [8–11], with each model reflecting determinate aspects of the whole biological system complexity. Here, we are primarily interested in the pausing and employ a generalization of a paradigmatic model of particle transport, the asymmetric simple exclusion process (ASEP, [12–14]) in the hydrodynamic limit [15]. The ASEP is a class of models of particles on a one-dimensional lattice, whose behaviour is chiefly determined by the rates at which the particles hop on the lattice. More specifically we require the rate profile function, which we refer to as $\tilde{\;p}$, to be spatially varying yet smooth as in [16,17], see also [18], thus making it possible to model this function by a Gaussian process (GP) [19]. Noticing the analogy between the PolII transport in the gene body and the particle hopping in the exclusion process, learning $\tilde{\;p}$ allows the study of the pausing dynamics in a gene. Importantly, we provide an inferential scheme to learn this rate function by Bayesian inference given real molecular biology data, assuming a prior on the profile function induced by a GP prior on a latent variable. In other words, integrating the dynamics defined by the rate $\tilde{\;p}$ generates transient time-course density profiles; we estimate $\tilde{\;p}$ given observed density profiles without explicitly inverting the system’s dynamics. Other models of PolII dynamics also leverage GPs for inference from biological data, with GPs representing transcriptional activity over time [20,21]. By contrast, the GP here describes a function of genomic position, with its minima corresponding to pausing regions. Due to its generality, our framework can be deployed to estimate the rate profiles of any one-dimensional transport problem.

The manuscript is organized as follows. Section 2.1 describes the biology of pausing and the next-generation sequencing (NGS) data types which are available to study it. Sections 2.2 and 2.3, respectively, discuss the ASEP as a mathematical model for transcription with pausing and a Bayesian inferential framework for model fitting. We present the results in §3 and conclude with a discussion in §4.

## 2. Model definition

### 2.1. Biological processes and data

RNA polymerases have a central role in the biology of transcription. We distinguish different classes of RNA polymerases, each having different structure and control mechanism. Bacteria and Archaea only have one RNA polymerase type. Eukaryotes have multiple types, of which RNA PolII is known to catalyse synthesis of protein-encoding RNA (messenger RNA or mRNA). In this paper, we describe mRNA transcription by PolII, but the inferential framework we present is general and can be extended to other transport phenomena. PolII binds to DNA upstream of the TSS, initiates the mRNA synthesis and then traverses the DNA downstream (elongation) until it pauses at a certain gene location, ready to respond to a developmental or environmental signal that instructs to resume the elongation. PolIIs are also found proximal to TSS in a so-called ‘poised’ state, which has not initiated synthesis of the mRNA chain. Poised and paused PolIIs can be differentiated as only paused PolIIs have a tail of nascent mRNA and are bound to transcription factor Spt5 [22]. The process terminates when the PolII reaches the TES and the transcribed mRNA is released. As a result of these steps, the output is modulated in both timing and intensity. However, many details, such as the pausing, are not well understood [5]. The presence of transcriptional pausing in eukaryotes is revealed by several assays based on NGS, which is widely used in molecular biology to study molecules involved in genic processes. In the PolII *ChIP-seq* assay, PolII-bound DNA is isolated by chromatin immunoprecipitation with a PolII antibody and is then subject to high-throughput sequencing. This provides a genome-wide view of the PolII binding sites for all forms of PolII, including both those poised or transcriptionally engaged and those which are bound to DNA and static. In ChIP-seq experiments, DNA fragments extracted from cells and associated with a specific protein (here polymerase) are amplified, sequenced and mapped to the reference genome, with fragments generally in the 150–300 Nt range [23] (while transcribing PolII covers less than 50 Nts of DNA [24]). This means that the precise locations of the individual proteins are not known and the assay only returns the overlap of reads from many different cells. For each genomic position, PolII ChIP-seq returns a signal as a proxy of polymerase occupancy.

For this study, we binned ChIP-seq reads from genomic ranges of selected genes (from cultured human cell lines, *Material and methods*) into 20 Nt bins, thus yielding coarse-grained read profiles (which we refer to as *y*) such as those illustrated in figure 1*b*. The number of these reads at a position *x* is proportional to the occupation probability *ϱ*(*x*). The proportionality factor, which depends on the number of cells used in the experiment and on further signal amplification intrinsic to the sequencing procedure, cannot be directly accessed with precision and is only known with substantial uncertainty [25].

Other methods available to study the pausing include but are not limited to *NET-seq*, where nascent mRNA chunks associated with immunoprecipitated PolII complexes are isolated and sequenced [26], *GRO-seq*, where RNAs recently transcribed only by transcriptionally engaged PolIIs are sequenced [27], and *PRO-seq*, which is similar to GRO-seq but reaches single-nucleotide resolution [28]. The evidences of PolII transport are particularly clear in time-course experiments, where sequencing data are collected over time following a perturbation. As an example, time-variant PRO-seq has been suggested to estimate pausing times in key peak regions [29]. A classical way to perturb these molecular dynamics is inhibiting the initiation by treating the cells with triptolide (Trp), which is a highly specific drug that blocks initiation [22,30]. This permits the PolII already engaged in transcription to progress further downstream the gene while new PolIIs are prevented from attaching, thus freeing upstream genomic regions as the run-on time progresses (figure 1*c*). Our approach consists of using the read profiles *y* as functions of *x*, collected at fixed run-on times *t*_1_, *t*_2_, *t*_3_ and *t*_4_ after treatment, to infer the dynamics. While Trp inhibits new initiation, poised PolII upstream of the TSS can still pass through it, enter the gene template and perform elongation immediately after Trp treatment [22,30]. To account for this, we also perform inference over Spt5 ChIP-seq data, where the poised polymerases are masked while those bound are detected [22].

These types of experiments reveal the presence of a flux of PolIIs, which is the signature of the non-equilibrium physics involved in the elongation process. The profile y* observed prior to the treatment corresponds to a non-equilibrium stationary state (NESS). Disrupting initiation with Trp yields a transient state, which evolves from y* until it settles down to a new NESS.

### 2.2. Mathematical model

The transport of particles on a one-dimensional lattice is a well-studied problem in mathematics and physics. Its basic features are captured by the ASEP [14], which defines the stochastic dynamics of interacting particles on a discrete lattice, which we take here to be a one-dimensional chain with open boundary conditions. Let the total number of lattice sites be *N*. The state of each site *i*, 1 ≤ *i* ≤ *N*, is characterized by the occupation number *n*_*i*_ such that *n*_*i*_ = 0 if the site is empty and *n*_*i*_ = 1 if it is occupied by a particle. The evolution proceeds in continuous time. A particle on site *i* < *N* hops rightward into the site *i* + 1 with rate *p*_*i*_, the transition being successful only if the site *i* + 1 is empty. Similarly, a particle on site *i* > 1 hops leftward into *i* − 1 with rate *q*_*i*_, if the site *i* − 1 is empty. Further, particles on the left (right) boundary site *i* = 1 (*i* = *N*) leave the lattice at rate *q*_1_ (*p*_*N*_), while particles are injected in the same boundary site at rate *p*_0_ (*q*_*N*+1_) if the site is empty. The constraint that a jump can occur only if the target is empty prevents the accumulation of more than one particle on a site and is generically referred to as the exclusion rule. This rule allows particle collision, which causes congestion when the particle density is sufficiently high and permits phase transitions between a low density, high density and a maximum current phase, even if the systems is one-dimensional [31]. Interestingly, based on theoretical considerations, it has been suggested that traffic-like congestion of PolIIs is important in transcription [32–34].

While the ASEP was originally proposed to model biopolymerization on nucleic acid templates [12,13], this and related models have been more recently applied to diverse problems, including protein translation [35–37], but also e.g. molecular motors [38] and pedestrian and vehicle traffic [39]. Applications to transcription incorporating disordered dynamics and obstacles (e.g. [40,41]) were also proposed. ASEP’s theoretical appeal is due to its analytical results representative of a large class of models [42,43] and a convenient mean-field treatment that yields the exact stationary solution [44]. In the context of transcription, particles entering site 1, moving along the chain and exiting from site *N* correspond to initiation, elongation and termination, respectively. In our setting, the lowest values of *p*_*i*_ correspond to genomic locations were elongation slows down.

The dynamics of the expected occupation of a single site *i* in the bulk are governed by the lattice continuity equation

2.1 $$\frac{d}{dt}E(n_{i}(t))=J^{\mathrm{left}}(t)-J^{\mathrm{right}}(t),$$

0 < *i* < *N*, where E denotes expectation value and *J*^left^(*t*) and *J*^right^(*t*) are the average flux of particles from site *i* − 1 to site *i* and from site *i* to site *i* + 1, respectively. These are subject to the exclusion rule and therefore obey

2.2 $$\begin{array}{rl} J^{\mathrm{left}}(t) & =p_{i-1}E(n_{i-1}(t)(1-n_{i}(t)))-q_{i}E(n_{i}(t)(1-n_{i-1}(t)))\\ \mathrm{and}\;\;\;J^{\mathrm{right}}(t) & =p_{i}E(n_{i}(t)(1-n_{i+1}(t)))-q_{i+1}E(n_{i+1}(t)(1-n_{i}(t))). \end{array}$$

In order to exactly solve these dynamics, second-order moments such as $E(n_{i}(t)n_{i+1}(t))$ need to be known. Under independence assumption, these moments are factorized, which in our case amounts to replacing equations (2.1) and (2.2) with

2.3 $$\begin{array}{rl} \frac{d}{dt}\phi_{i}(t) & =p_{i-1}\phi_{i-1}(t)(1-\phi_{i}(t))-p_{i}\phi_{i}(t)(1-\phi_{i+1}(t))\\ & \;+q_{i+1}\phi_{i+1}(t)(1-\phi_{i}(t))-q_{i}\phi_{i}(t)(1-\phi_{i-1}(t)), \end{array}$$

where we used $\phi_{i}(t)\;:=E(n_{i}(t))$ to lighten the notation. In other words, equations (2.3) define the so-called mean-field dynamics of the asymmetric exclusion process, which are known to approximate well the true dynamics in many contexts, predict crucial features such as dynamical phase-transitions, and ease mathematical treatment [31,44,45]. With open boundaries,

2.4 $$\frac{d}{dt}\phi_{1}(t)=p_{0}(1-\phi_{1}(t))-p_{1}\phi_{1}(t)(1-\phi_{2}(t))-q_{1}\phi_{1}(t)+q_{2}\phi_{2}(t)(1-\phi_{1}(t)),$$

and

2.5 $$\begin{array}{rl} \frac{d}{dt}\phi_{N}(t) & =p_{N-1}\phi_{N-1}(t)(1-\phi_{N}(t))\\ & \;-p_{N}\phi_{N}(t)+q_{N+1}(1-\phi_{N}(t))-q_{N}\phi_{N}(1-\phi_{N-1}). \end{array}$$

To match the available data that is coarse grained (figure 1*b*), instead of considering particles individually we rely on their hydrodynamics description, which is obtained as follows. We assume Euler scaling with constant *a* and let *a* → 0, *N* → ∞, with *L* := *N a* held finite. We define the functions $\varrho \;:\;R^{2}\to R_{0}^{+}$, $\tilde{p}\;:\;R\to R_{0}^{+}$ and $\tilde{q}\;:\;R\to R_{0}^{+}$ such that they are analytic and bounded on ]0, *L*[ × ]0, ∞[, ]0, *L*[ and ]0, *L*[, respectively, and

2.6 $$\begin{array}{rl} \phi_{i}(t) & =\varrho ((i-1)a,t),\\ ap_{i} & =\tilde{p}((i-1)a)\\ \mathrm{and}\;\;aq_{i} & =\tilde{q}((i-1)a). \end{array}$$

We further assume that the left and right jump rates satisfy $\tilde{q}(x)=b\tilde{\;p}(x)$, ∀x∈[0,L], with 0 ≤ *b* < 1, where *b* governs the relative strength of the non-equilibrium driving forces. The case *b* = 0 corresponds to a *totally asymmetric exclusion process* (TASEP), while the limit case *b* = 1 corresponds to the *symmetric exclusion process*. Intermediate values 0 < *b* < 1 correspond to settings where the particles can jump in both directions, but are driven rightwards on average. A continuum-limit counterpart of equations (2.2), as derived in [16,18], is

2.7 $$\begin{array}{rl} J(x,t) & =\tilde{p}(x)\varrho \left(x-\frac{a}{2},t\right)\left(1-\varrho \left(x+\frac{a}{2},t\right)\right)\\ & \;-\tilde{q}(x)\varrho \left(x+\frac{a}{2},t\right)\left(1-\varrho \left(x-\frac{a}{2},t\right)\right), \end{array}$$

which, using first-order Taylor expansion, yields

2.8 $$J(x,t)\approx (\tilde{p}(x)-\tilde{q}(x))\varrho (x,t)(1-\varrho (x,t))-\frac{a}{2}(\tilde{p}(x)+\tilde{q}(x))\frac{\partial }{\partial x}\varrho (x,t).$$

To lighten the mathematical notation, we define the two quantities

2.9 $$\begin{array}{rl} \lambda (x) & \;:=(\tilde{p}(x)-\tilde{q}(x))=\tilde{p}(x)(1-b)\\ \mathrm{and}\;\;\;\nu (x) & \;:=\frac{a}{2}(\tilde{p}(x)+\tilde{q}(x))=\frac{a}{2}\tilde{p}(x)(1+b) \end{array}$$

their ratio is constant in *x*, viz., *ν*(*x*)/*λ*(*x*) = *a*/2 (1 + *b*)/(1 − *b*), which equals *a*/2 in the totally asymmetric case.

Substituting (2.8)–(2.9) into the continuity equation

2.10 $$\frac{\partial }{\partial t}\varrho (x,t)=-\frac{\partial }{\partial x}J(x,t),$$

which is the hydrodynamics limit of equation (2.1), gives the nonlinear partial differential equation

2.11 $$\frac{\partial }{\partial t}\varrho (x,t)=-\frac{\partial }{\partial x}\{\lambda (x)\varrho (x,t)(1-\varrho (x,t))-\nu (x)\frac{\partial }{\partial x}\varrho (x,t)\},$$

which can be linearized to

2.12 $$\frac{\partial }{\partial t}u(x,t)=\frac{\tilde{\;p}(x)}{2}\left\{a^{2}(1+b)\frac{\partial^{2}}{\partial^{2}x}u(x,t)-\frac{{\left(1-b\right)}^{2}}{1+b}u(x,t)\right\}$$

by means of a generalization of the Cole–Hopf transform (electronic supplementary material, appendix A and [18,46,47]).

In transcription, the particle flux is left to right. While PolIIs can backtrack few Nts under certain circumstances [48–50], this phenomenon is overall minor and is not observable at our ChIP-seq resolution. Therefore, we assume *b* = 0 and focus on the inference of the net forward rate profile $\tilde{\;p}(x)$. For simplicity we also set *a* = 1, arguing that our considerations remain valid with such a choice. The required boundary values *ϱ*(0, *t*), *ϱ*(*L*, *t*) and *ϱ*(*x*, 0), and the numerical scheme used to integrate equation (2.12) are detailed in electronic supplementary material, appendices A and B.

**Figure 2.**
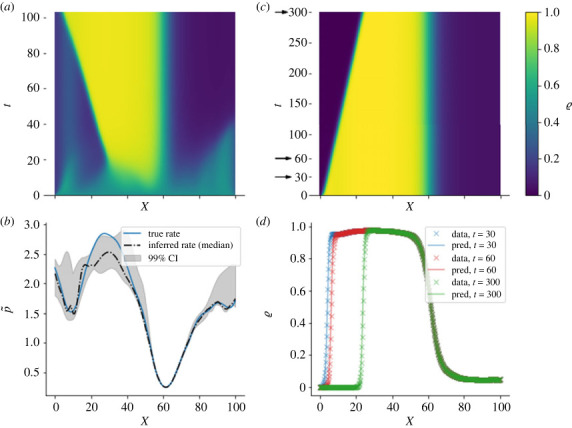
Simulation study. (*a*) A non-equilibrium stationary state (NESS) of profile ϱ*(*x*) is obtained integrating the hydrodynamic TASEP with open boundaries, initial density profile ϱ(x,0)=0.5 ∀x∈[0,L] and chosen rate profile (solid line in (*b*)). (*b*) True rate profile (solid line) and inferred rate profile (dash-dot line); the shaded area is 99% credible interval (CI). (*c*) Integrating the same dynamics with initial profile ϱ*(*x*) and no-influx boundary conditions shows that the density decreases in proximity of the left boundary, similar to ChIP-seq readings followed by Trp treatment; the density profiles corresponding to times 30, 60 and 300 and used for inference are marked by arrows. (*d*) The posterior predictive samples (solid lines) are in excellent agreement with the extracted density profiles (cross markers); the posterior predictive dispersion is of the order of the line width, see also electronic supplementary material, figure S3

Integrating equation (2.11) with boundary conditions analogous to equations (2.4) and (2.5) and initial density *ϱ*(*x*, 0) > 0 yields a NESS for large *t*, characterized by a non-vanishing average flux and a density profile ϱ*(*x*) which is invariant in time. Setting the latter as initial condition and further integrating with no inward particle flux (*p*_0_ = *q*_*N*_ = 0) produces a transient state that mimics the evolution of the PolII profile after Trp treatment until the density profile vanishes. This is illustrated, for a choice of boundary values and jump rate profile, in figure 2, which also includes the result of the inference process described in the next sections.

### 2.3. Bayesian framework

We fit the model to real-world data by means of a Bayesian approach leveraging its ability to explicitly encode prior hypotheses about the quantities we wish to infer [51]. We are interested in the forward rate profile $\tilde{\;p}$. As this is required to be analytic and non-negative, it is convenient to assume a GP [19] functional prior on a latent variable *f* and induce a prior on $\tilde{\;p}$ using a sigmoid link function of *f*, such that $\tilde{\;p}={\tilde{\;p}}_{\max}/(1+\exp(-f))$, which further imposes an upper bound ${\tilde{\;p}}_{\max}$ to $\tilde{\;p}$. The GP prior here defines a distribution over real valued *C*^1^ functions in R, where any finite set of function evaluations *f*(*x*) has multivariate normal distribution with mean *m* and covariance kernel $k(x,x^{\prime };\sigma_{f}^{2},l)=\sigma_{f}^{2}\exp(-{\left(x-x^{\prime }\right)}^{2}/(2\;l^{2}))$, $x,x^{\prime }\in R$. In practice, the GP is evaluated at the positions *x*_*i*_, i=1,…,n, where it is equivalent by definition to f∼N(m,K), a multivariate normal random variable with mean *m* and covariance matrix **K**(*σ*_*f*_, *l*) induced by the kernel.

The observations are organized into a collection of values $y={\left\{y_{ij}\right\}}_{i=1,2,\ldots ,\text{n},j=1,2,\ldots ,\text{t}}$, where the subscripts indicate that an observation is taken at position *x*_*i*_ and time *t*_*j*_. As the values of *x*_*i*_ do not necessarily coincide with the bin centres of ChIP-seq data, we used simple linear interpolation to estimate the data at intermediate coordinates. We assume that the observed values depend on a multiplicative factor and also include an additive error term $\epsilon ∼N(0,\sigma_{\epsilon })$. This can be written in terms of the equation $\kappa y_{ij}=\varrho (x_{i},t_{j})+\epsilon$, where *κ* is the inverse of the amplification factor. The likelihood $P(y|f,\sigma_{\epsilon },\kappa )$ satisfies

2.13 $$\log P(y|f,\sigma_{\epsilon },\kappa )=-\frac{1}{2\sigma_{\epsilon }^{2}}\sum_{i=1}^{\text{n}}\sum_{\;j=1}^{\text{t}}(\varrho (x_{i},t_{j};f)-\kappa y_{ij})-\frac{n}{2}\log(2\pi \sigma_{\epsilon }^{2}),$$

where we made explicit that *ϱ* depends on **f**. For the hierarchical parameters $(m,\sigma_{\epsilon },\kappa ,\sigma_{f},l)=\;:\;\theta$ we assume a scaled sigmoid Gaussian prior probability $P(m,\sigma_{\epsilon },\kappa ,\sigma_{f},l)$ such that

2.14 $$\theta =\theta_{\min}+\frac{\left(\theta_{\max}-\theta_{\min}\right)}{1+\exp(-\xi )},\;\xi ∼N(\mu_{\xi },1\sigma_{\xi }),$$

where $\theta_{\min}\;:=(m_{\min},{\sigma_{\epsilon }}_{\min},\kappa_{\min},{\sigma_{\;f}}_{\min},l_{\min})$, $\theta_{\max}\;:=(m_{\max},{\sigma_{\epsilon }}_{\max},\kappa_{\max},{\sigma_{\;f}}_{\max},l_{\max})$ and $\left(\mu_{\xi },\sigma_{\xi }\right)$ are referred to as hyperparameters. Prior distributions are chosen to pull Markov chain Monte Carlo (MCMC) samples away from inappropriate results that are consistent with the likelihood but would not be consistent with domain knowledge [51]. By using scaled sigmoid Gaussian prior probability bounded by *θ*_min_ and *θ*_max_, we only search for solutions constrained in an appropriate interval [52]. By virtue of the Bayes theorem the joint posterior probability for *θ* and **f** satisfies

2.15 $$P(f,m,\sigma_{\epsilon },\kappa ,\sigma_{f},l|y)\propto P(y|f,\sigma_{\epsilon },\kappa )P(f|m,\sigma_{f},l)P(m)P(\sigma_{\epsilon })P(\kappa )P(\sigma_{f})P(l),$$

which we draw random samples from by MCMC sampling, more specifically block Gibbs sampling with elliptical slice sampling at each block [52,53] (electronic supplementary material, appendix C). Equation (2.15) expresses the distribution of parameters given the observed data **y** and completes the definition of the model. It is worth noting that evaluating the likelihood also requires computing *ϱ* by integrating equation (2.11) with initial condition *ϱ*(*x*, 0) = κy*(*x*), ∀x∈[0,L].

## 3. Results

We first consider simulated data from a given profile of length *L* = 100 obtained from GP drawn with parameters $\left(l,\sigma_{f},m,{\tilde{\;p}}_{\max})=(7.32,0.67,0.29,3\right)$. We integrate the dynamics with NESS initial profile (obtained by fixing the boundary conditions to *ϱ*(0, *t*) = *ϱ*(*L*, *t*) = 0.5, ∀t) and no-influx boundary conditions (figure 2*a*,*b*). The chosen rate profile shows a local minimum close to the left boundary, which yields a minor local perturbation in the density, and a global minimum around *x* ≈ 60, whose effect propagates along the lattice and acts as a major bottleneck, which separates a low-density phase downstream from a high-density phase upstream. These minima correspond to regions where particles slow down or pause for an exponentially distributed amount of time. As the particles leave the system through the right boundary and are not replenished by the influx through the left boundary, the region upstream of the bottleneck is emptied by a reverse wavefront.

For the purpose of testing whether we are able to recover the rate profile from time-course observations, we extract density profiles **y** at times (*t*_1_, *t*_2_, *t*_3_) = (30, 60, 300) and set the hyperparameters *θ*_min_, *θ*_max_ and $\left(\mu_{\xi },\sigma_{\xi }\right)$ to (0, 0, 0.8, 0, 0), (2, 10, 1.2, 1, 10) and (0, 1), respectively. With these settings and data, we generated 10^4^ MCMC samples targeting the posterior (2.15), discarding the first 2 × 10^3^ as burn-in, demonstrating that the fitting procedure is able to capture the location of both the major and minor minima of the generative model, as well as the overall elongation rate (figure 2*b*). It is worth noting that the integrated density profile in figure 2*c*,*d* displays a very small effect of the first local minimum (minor dip, captured only by time-course profiles at *t*_1_ and *t*_2_); this is reflected in relatively wide credible intervals for the inferred rate profile (grey ribbon in figure 2*b*). On the other hand, the rate at the bottleneck is inferred with very high confidence. The covariance hyperparameters *l* and *σ*_*f*_ control how quickly the rate changes over *x*; these were slightly misestimated to 6.86 (95% CI 4.63–7.16) and 0.76 (95% CI 0.75–0.82), respectively, thus suggesting that increased wobbling in the rate profile is tolerated; minor patterns in the rate profile are in fact smoothed out and are essentially not identifiable in the density profiles obtained by integration (see electronic supplementary material, figure S3). The difficulty of sampling covariance hyperparameters is also addressed, e.g. in [52]. The predicted transient density profiles at *t* = 30, 60, 300 also are in very good agreement with the input data (figure 2*d*; in fact, all sampled rate profiles yield similar time-course density profiles despite wide CIs in certain regions (see also electronic supplementary material, figure S3).

Applying this method to real-world data requires setting the value of ${\tilde{\;p}}_{\max}$ to an upper limit of prior expectations on the elongation rate. As this has been estimated at around 2 × 10^3^ Nt min^−1^ in previous studies [30], we set ${\tilde{\;p}}_{\max}=6\times 10^{3}\;\mathrm{Nt}\;\min^{-1}$ as an arguably safe upper bound. Literature results can be also used to set bounds on the prior for *κ*, which regularizes the estimation problem [19]. From cultured human cell lines, the total number P of bound PolII molecules per cell is estimated to be between P_min_ = 11 × 10^5^ and P_max_ = 18 × 10^5^ [54]. This is related to the total number *Y* of ChIP-seq counts by P = *κY*. Based on these heuristic considerations, we set *κ*_min_ = P_min_/*Y* and *κ*_max_ = P_max_/*Y*. All remaining hyperparameters were set identical to the previous simulation experiment.

**Figure 3.**
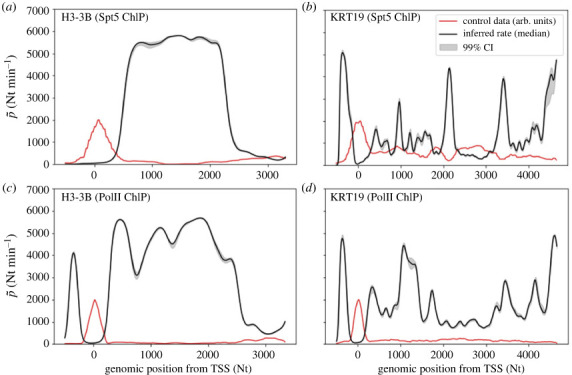
Inferred rate profiles from Spt5 ChIP-seq (*a*,*b*) and PolII ChIP-seq (*c*,*d*) for genes *H3-B3* (*a*,*c*) and *KRT19* (*b*,*d*) (black lines are posterior medians, shaded areas are 99% credible intervals), with the latter gene showing a distinctive jagged profiles. Both genes show the lowest rates in proximity of the transcription starting site (TSS). Red lines are unperturbed ChIP-seq signals in arbitrary units (arb. units).

**Figure 4.**
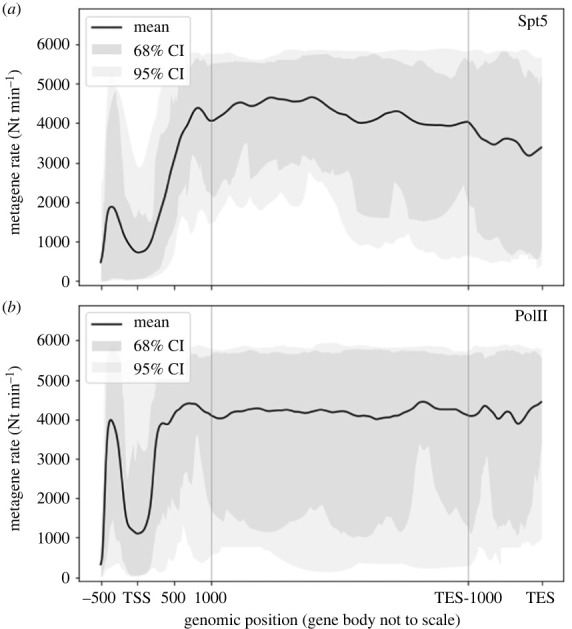
Metagene rates from PolII ChIP-seq (*a*) and Spt5 ChIP-seq (*b*) data. By construction, the metagene analysis conserves the length scale only in proximity of TSS and TES. Upstream of TSS the Spt5 ChIP-seq yields lower average rate than PolII ChIP-seq, as this assay does not detect PolII poised to move downstream.

**Figure 5.**
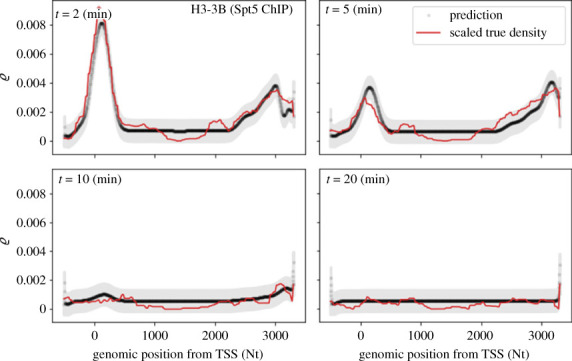
Predicted density profiles for gene *H3-3B* from Spt5 ChIP-seq 2, 5, 10 and 20 min after Trp treatment as samples from posterior predictive distribution. Grey area is 95% credible interval due to noise model.

The results from different genes show a variety of rate profiles which share similar patterns (figure 3). The most important observation is that, in all genes considered, the rates vary strongly with the genomic position, with local minima corresponding to regions where PolIIs slow down or pause. In order to look for average patterns, it is desirable to aggregate data from all genes. As genes have different lengths (which in our sample range from 16 680 to 59 880 Nts), we stretch all the rate profiles in the region from TSS + 1000 to TES − 1000 Nts to the same support length and then average over the genes at each position. This yields the summaries illustrated in figure 4, which we refer to as *metagene* rates and are akin to the so-called metagene profiles [55]. Rates are typically lower near the TSS than in the gene body, where elongation approaches its highest rate. The behaviour in proximity of the TES is less definite, with rates varying several fold among the different genes. At the TSS the rate typically dips down consistently with the presence of strong and widespread pausing in this region. Further downstream in the gene body the rate increases to its highest average value. While the dip is evident in both Spt5 and PolII results, it is worth noting that upstream of the TSS the average rate inferred from PolII data is higher than that from Spt5. We argue that this difference is due to the fact the former also include poised PolIIs which are not strongly bound to the template and can quickly move towards the TSS before being engaged in transcription. A by-product of the fitting procedure is the estimate of the occupation density *ϱ*(*x*, *t*) = *κy*(*x*, *t*), as illustrated in figure 2*d* for the simulation experiment and figure 5 and electronic supplementary material, figures S4–S6 for selected genes. The predicted densities are typically very low (total predicted number of PolIIs in a gene is of the order of 10^−1^), thus suggesting that crowding and congestion of PolIIs into a gene might not be substantial even proximal to rate minima.

## 4. Discussion

We developed a general Bayesian framework to study the dynamics of a one-dimensional transport model given time-resolved density profiles. The general problem addressed here is the identification of the PDE parameters that best describe data as a subset of the true PDE solution (see, e.g. [52,56–58] and references therein). We focused on the hydrodynamic TASEP with smoothly varying jump rates (which are the parameters to be inferred) as a paradigmatic and well-characterized model of transport. By means of its application to ChIP-seq time-course data, we inferred the rate of PolII elongation as a function of the genomic position in selected genes. This rate is not constant but varies within the gene body. It typically dips down nearby the TSS, confirming widespread pausing in this region, while in the bulk the rate also varies between genes. Low predicted densities suggest that the pausing did not cause congestion or crowding. This is an important observation, as factor crowding has been experimentally observed and associated with regulated gene expression in synthetic and mammalian cell systems [59–63]. Our analysis supports the view that this phenomenon does not happen between PolIIs bound to the gene but probably occurs in suspension in the nucleoplasm, as described e.g. in [61,63–67].

The inference here is complicated by the high dimensionality of the parameter space (which grows as the genes’ length increases). We addressed this by assuming a GP latent prior for the jump-rate profile and using elliptic slice sampling as an appropriate MCMC algorithm. The sampling requires multiple evaluations of the likelihood of equation (2.13). This in turns requires numerically integrating equation (2.11), which is also slower in longer genes (require larger integration grids, see electronic supplementary material, appendix B).

This study of molecular dynamics is also subject to limitations. While ChIP-seq is a widely used assay to quantify the abundances of DNA-bound PolII, studies suggest that it has limited resolution (between 150 and 300 Nts) and might be subject to technical issues [23]. Most importantly ChIP-seq profiles are obtained from the aggregation of sequencing reads from many cells, which hides variation within the cell population. The transcription of mRNA is a very complex process and it may be interesting to include features not encoded in the model used for this study. Other TASEP variants, such as those incorporating non-Markovian jump dynamics [68,69] or Langmuir kinetics [70], are relevant for the modelling of PolII recycling and its early detachment from DNA [63,71]. An assumption of the TASEP is that particles stay in a site for an exponentially distributed waiting time. Variants of TASEP in which defects appear and disappear randomly on any site (and thus slow down the movement of particles or even block it completely) have been introduced in physics literature and can account for occasionally long pausing times [40,41] (see also [68,69]), with defect dynamics representing the effects of pausing and elongation factors. Modelling advancements that combine the site-specific pausing with long pausing times and extended particle size, supplemented by an appropriate inference scheme such as the one presented here, would be an important additional potential area for research and application. Potential extensions of our work also include estimation of the parameters that encode the system’s size and asymmetry (*a* and *b*, respectively) and the boundary values. Statistical mechanics literature is rich in quantitative studies of TASEPs with particles that occupy more than one lattice site [72–74], some generalized to include site-dependent elongation rates or localized defects [37,75–77]. These studies have been used to describe protein translation and could be useful to predict PolII-size effects in gene expression, although, with genes much longer than PolIIs, ChIP-seq limited resolution, and very low PolII coverage density, the observable correction would arguably be minor. In fact, including more features plausibly requires sequencing assays of higher resolution than ChIP-seq and comes at the cost of increased computational burden and decreased tractability. Conversely, the chosen TASEP with smoothly varying jump rates is simple and yet is able to reveal PolII elongation slowing down and speeding up at certain genomic locations. Due to its generality, our approach also serves as a template for future studies seeking to shed light on complex transport phenomena.

## 5. Material and methods

Spt5 and PolII ChIP-seq data mapped to the hg19 University of California at Santa Cruz human genome were downloaded from Gene Expression Omnibus (GEO, http://www.ncbi.nlm.nih.gov/geo), accession number GSE117006. We filtered the list of genes from the reference genome to only contain those with unique gene symbols on chromosomes 1–22 and X, thus excluding alternatively spliced genes. Hg19 gene coordinates were flanked 500 Nts upstream of the TSS in order to include poised PolII. The 20 non-overlapping genes with the highest coverage of Spt5 ChIP-seq reads were selected. All simulation codes are written in c++ and Python (v. 3.7.1), with the PDE solver using Numba JIT compiler (v. 0.41.0) [78] (https://github.com/mcavallaro/dTASEP-fit).

## Data Availability

Supplementary material is available online [79].
